# *Xanthomonas citri* pv. *viticola* Affecting Grapevine in Brazil: Emergence of a Successful Monomorphic Pathogen

**DOI:** 10.3389/fpls.2019.00489

**Published:** 2019-04-18

**Authors:** Marisa A. S. V. Ferreira, Sophie Bonneau, Martial Briand, Sophie Cesbron, Perrine Portier, Armelle Darrasse, Marco A. S. Gama, Maria Angélica G. Barbosa, Rosa de L. R. Mariano, Elineide B. Souza, Marie-Agnès Jacques

**Affiliations:** ^1^Departamento de Fitopatologia, Universidade de Brasília, Brasília, Brazil; ^2^IRHS, INRA, AGROCAMPUS-Ouest, SFR4207 QUASAV, Université d’Angers, Beaucouzé, France; ^3^Laboratório de Fitobacteriologia, Departamento de Agronomia, Universidade Federal Rural de Pernambuco, Recife, Brazil; ^4^Embrapa Semiarido, Petrolina, Brazil

**Keywords:** MLVA, MLSA, *Vitis vinifera*, grapevine bacterial canker, *Xanthomonas campestris* pv. *viticola*

## Abstract

The pathovar *viticola* of *Xanthomonas citri* causes bacterial canker of grapevine. This disease was first recorded in India in 1972, and later in Brazil in 1998, where its distribution is currently restricted to the northeastern region. A multilocus sequence analysis (MLSA) based on seven housekeeping genes and a multilocus variable number of tandem repeat analysis (MLVA) with eight loci were performed in order to assess the genetic relatedness among strains from India and Brazil. Strains isolated in India from three related pathovars affecting Vitaceae species and pathogenic strains isolated from *Amaranthus* sp. found in bacterial canker-infected vineyards in Brazil were also included. MLSA revealed lack of diversity in all seven genes and grouped grapevine and Amaranthus strains in a monophyletic group in *X. citri*. The VNTR (variable number of tandem repeat) typing scheme conducted on 107 strains detected 101 haplotypes. The total number of alleles per locus ranged from 5 to 12. A minimum spanning tree (MST) showed that Brazilian strains were clearly separated from Indian strains, which showed unique alleles at three loci. The two strains isolated from symptomatic *Amaranthus* sp. presented unique alleles at two loci. STRUCTURE analyses revealed three groups congruent with MST and a fourth group with strains from India and Brazil. Admixture among populations were observed in all groups. MST, STRUCTURE and e-BURST analyses showed that the strains collected in 1998 belong to two distinct groups, with predicted founder genotypes from two different vineyards in the same region. This suggest that one introduction of grape planting materials contaminated with genetically distinct strains took place, which was followed by pathogen adaptation. Genome sequencing of one Brazilian strain confirmed typical attributes of pathogenic xanthomonads and allowed the design of a complementary VNTR typing scheme dedicated to *X. citri* pv. *viticola* that will allow further epidemiological survey of this genetically monomorphic pathovar.

## Introduction

*Xanthomonas citri* pv. *viticola*, the causal agent of grapevine bacterial canker, was first described in India as *Pseudomonas viticola* sp. nov. (Nayudu, 1972). For many years, its occurrence was restricted to India and regarded as a disease of secondary importance until outbreaks in the late 1980’s (Chand and Kishun, 1990). In 1998, a new disease was reported affecting vines of *Vitis vinifera* cultivar Red Globe in the irrigated areas of the São Francisco River valley in Pernambuco and Bahia states, northeastern Brazil. This region accounts for a significant percentage of table grape production in Brazil. Disease symptoms were leaf spots and cankers observed on stems, twigs and petioles. The causal agent was identified through biochemical and pathogenicity tests as *Xanthomonas* *campestris* pv. *viticola*. Additionally, rep-PCR fingerprinting analysis of strains collected in Brazil showed highly similar profiles to the Indian pathotype strain (NCPPB 2475) (Trindade et al., 2005). Infected grapevines were later detected in other states in Brazil (Halfeld-Vieira and Nechet, 2006; Rodrigues Neto et al., 2011), and eradication procedures were adopted since the pathogen is of quarantine significance and subjected to regulatory measures. Besides India and Brazil, the pathogen has been reported in Africa in 2005 (Midha and Patil, 2014). The pathogen may disseminate by infected propagating material and an association with seeds and berries was demonstrated suggesting systemic colonization and spread (Tostes et al., 2014). Natural hosts of pathovar *viticola* are *V. vinifera* varieties. Nayudu (1972) also reported natural infection of *Azadirachta* *indica* (neem, Meliaceae) and *Phyllanthus maderaspatensis* (Euphorbiacae), which may represent alternative sources of inoculum for infection of grapevines. Plants in the Anacardiaceae family, such as mango tree (*Mangifera indica*) have also been described as potential hosts through inoculation (Chand and Kishun, 1990). In Brazil, some weed species belonging to the genera *Alternanthera*, *Amaranthus*, *Glycine*, and *Senna* have been identified as potential alternative hosts as well (Peixoto et al., 2007).

Diagnosis of grapevine bacterial canker is based on symptom observation followed by bacterial isolation and identification tools, including induction of a hypersensitive reaction (HR) on tomato leaves, pathogenicity tests on susceptible varieties, serology with polyclonal antibodies and/or molecular identification tests based on PCR (Trindade et al., 2005, 2007; Gama et al., 2018). Primers have been designed on partial sequences of the *hrp* cluster that differentiate *Xanthomonas* strains at both pathovar and species levels (Leite et al., 1994) and were shown to be useful for detection and identification of pathovar *viticola* in culture and plant tissue (Trindade et al., 2007).

The pathovar *viticola*, a non-pigmented xanthomonad, has been referred as *Xanthomonas campestris sensu lato* since it was not included in the *Xanthomonas* reclassification study of Vauterin et al. (1995). Sequence analysis of the housekeeping gene gyrase B (*gyrB*) for over 200 xanthomonads, including 67 poorly characterized pathovars of *X. campestris*, placed the pathotype strain from India and three pathovars associated with hosts formerly classified in the genus *Vitis*, in the *X. citri* subsp. *citri* clade (Parkinson et al., 2009), along with several members of group 9.5 of *X. axonopodis*, such as pathovars *citri*, *glycines*, and *mangiferaeindicae* (Ah-You et al., 2009; Mhedbi-Hajri et al., 2013). Coherently, it was recently included in the newly proposed *X. citri* species that encompasses the so-called 9.5 and 9.6 groups (Rademaker et al., 2005; Constantin et al., 2016). A taxonomic reposition as *X. citri* pv. *viticola* comb. nov. has been proposed (Gama et al., 2018). In addition, phylogenomic analysis revealed that several pathovars, including pathovar *viticola*, form a monophyletic cluster and belong to one species, *X. citri* (Bansal et al., 2017).

Multilocus variable number of tandem repeats analysis (MLVA) is a high-resolution method for monitoring epidemics and assessing population structure and diversity for many bacterial species. A typical variable number of tandem repeats (VNTR) locus shows large range of copy numbers even among highly related bacterial strains (Jackson, 2010). MLVA has been used as a typing tool in outbreaks of numerous human and animal pathogens, but also in food microbiology such as the winemaking process (Claisse and Lonvaud-Funel, 2012). For human pathogens of medical interest, it has been regarded as a powerful tool for outbreak detection and source tracing in several European countries (Lindstedt et al., 2013). Resources for discovery of polymorphic loci such as VNTR databases are available for free access (Chang et al., 2007). For plant associated bacteria, several MLVA schemes have been described for important pathogens including several species and pathovars of *Xanthomonas* spp. such as *X.* *citri* pv. *citri* (Bui Thi Ngoc et al., 2009; Pruvost et al., 2014; Leduc et al., 2015); *X. oryzae* (Poulin et al., 2015); *X. arboricola* (Cesbron et al., 2014; Essakhi et al., 2015; López-Soriano et al., 2016); *X. fragariae* (Gétaz et al., 2018); and *X. axonopodis* pv. *manihotis* (Arrieta-Ortiz et al., 2013). VNTR typing has been recognized as the best tool to type recently emerged bacteria with limited genetic diversity and to better understand their patterns of long-distance dissemination (Bühlmann et al., 2013; Cunty et al., 2015; Nakato et al., 2018).

The objectives of this study were to assess the genetic relatedness among *Xanthomonas* *citri* pv. *viticola* strains from India and Brazil, related pathovars (pv. *vitiscarnosae*, *vitistrifoliae*, *vitiswoodrowii*) affecting other host plants in the family Vitaceae and three pathogenic strains from *Amaranthus* sp. collected close to bacterial canker-infected grapevines in Brazil. We conducted the characterization of pathovar *viticola* strains based on a concatenated sequence of seven housekeeping genes (MLSA) to allow comparisons with strains from other pathovars. A MLVA typing scheme with eight VNTR loci derived from *X. citri* pv. *citri* was validated with 107 strains of pathovar *viticola* and used to assess the genetic structure of this pathovar in Brazil. Genome sequencing of one Brazilian strain confirmed typical attributes of pathogenic xanthomonads and allowed the design of a complementary VNTR typing scheme dedicated to *X. citri* pv. *viticola* that will allow further epidemiological survey of this genetically monomorphic pathovar.

## Materials and Methods

### Bacterial Strains

A collection of 102 strains isolated from grapevine (*Vitis vinifera*) and three strains isolated from symptomatic *Amaranthus* sp. plants growing in bacterial canker-infected vineyards in Brazil were used in this study (Table 1). These strains were isolated over a period of 14 years (1998–2012). The pathovar *viticola* pathotype strain from India, two other Indian strains from grapevine (*V. vinifera*) and the pathotype strains (CFBP 7658, CFBP 7659, and CFBP 7657) of the three pathovars affecting other species in the Vitaceae family (*vitiscarnosae*, *vitistrifoliae*, and *vitiswoodrowii*, respectively) were also included in this study. These six strains were isolated in India from 1951 to 1972 (Table 1). The strains isolated from grapevine plants in Brazil were previously identified by PCR with specific primers targeting a 240 bp-sequence of the *hrcN* *(hrpB)* gene (Trindade et al., 2007) and tested for hypersensitive response (HR) induction on tomato and/or pathogenicity on a susceptible grapevine cultivar.

**Table 1:** *Xanthomonas* strains used in this study.

Strain	Other collections	Host cultivar	Country of origin	Location^a^	Year of isolation	Pathogenicity	HR tomato	PCR^b^
CFBP 7660	NCPPB 2475, LMG 965, ICMP 3351	Anab-e-Shahi	India	Andhra Pradesh	1969	+	+	+
CFBP 7691	NCPPB 2614, LMG 966, ICMP 3865	na^c^	India	na	1972	nd^d^	nd	nd
CFBP 7694	NCPPB 3642	na	India	na	1990^e^	-	nd	nd
CFBP 5869	IBSBF 1385	Italia	Brazil	Teresina, PI	1998	+	+	+
CFBP 7675	ICMP 13704, IBSBF 1376	Red Globe	Brazil	Petrolina, PE	1998	nd	nd	nd
CFBP 7676	ICMP13706, IBSBF 1386	Ribier	Brazil	Teresina, PI	1998	+	+	+
CFBP7764	P1S6	Red Globe	Brazil	Petrolina, PE	2012	+	+	+
482	IBSBF 2598		Brazil	Boa Vista, RR	2006	+	nd	+
4562	IBSBF 1507	Red Globe	Brazil	Petrolina, PE	1999	+	nd	+
1184		Red Globe	Brazil	Petrolina, PE	1998	+	+	+
1186		Red Globe	Brazil	Petrolina, PE	1998	+	+	+
1187		Red Globe	Brazil	Petrolina, PE	1998	+	+	+
1189		Red Globe	Brazil	Petrolina, PE	1998	+	+	+
1191		Italia	Brazil	Petrolina, PE	1998	+	+	+
1192		Italia	Brazil	Petrolina, PE	1998	+	+	+
1193		Red Globe	Brazil	Petrolina, PE	1998	+	+	+
1194		Red Globe	Brazil	Petrolina, PE	1998	+	+	+
1195		Red Globe	Brazil	Petrolina, PE	1998	+	+	+
1205		Italia	Brazil	Sobradinho, BA	2000	+	+	+
1226		Thompson Seedless	Brazil	Petrolina, PE	2001	+	+	+
1299		Thompson Seedless	Brazil	Juazeiro, BA	2004	+	+	+
1303		Sugraone	Brazil	Petrolina, PE	2005	nd	+	+
1307		Sugraone	Brazil	Petrolina, PE	2005	+	+	+
1309		Sugraone	Brazil	Petrolina, PE	2005	+	+	+
1315		Red Globe	Brazil	Petrolina, PE	2005	+	+	+
1316		Red Globe	Brazil	Juazeiro, BA	2005	nd	+	+
1318		BRS Morena	Brazil	na	2006	+	+	+
4779B		Red Globe	Brazil	Cianorte, PR	2009	+	+	+
A2		Red Globe	Brazil	Petrolina, PE	2010	+	+	+
A11		Thompson Seedless	Brazil	Petrolina, PE	2010	nd	nd	+
A12		Thompson Seedless	Brazil	Petrolina, PE	2010	nd	nd	+
AR1		Red Globe	Brazil	Petrolina, PE	2012	nd	+	+
AR2		Red Globe	Brazil	Petrolina, PE	2012	nd	nd	+
TR1		Red Globe	Brazil	Petrolina, PE	2012	nd	nd	+
TR3		Red Globe	Brazil	Petrolina, PE	2012	nd	+	+
P1S5		Red Globe	Brazil	Petrolina, PE	2012	nd	nd	+
P1S9		Red Globe	Brazil	Petrolina, PE	2012	nd	+	+
P1S12		Red Globe	Brazil	Petrolina, PE	2012	nd	nd	+
P1S16		Red Globe	Brazil	Petrolina, PE	2012	nd	nd	+
P2S1		Red Globe	Brazil	Petrolina, PE	2012	nd	nd	+
P2S2		Red Globe	Brazil	Petrolina, PE	2012	+	nd	+
P2S4		Red Globe	Brazil	Petrolina, PE	2012	+	+	+
P2S6		Red Globe	Brazil	Petrolina, PE	2012	nd	+	+
P2S7		Red Globe	Brazil	Petrolina, PE	2012	nd	nd	+
RS2		Red Globe	Brazil	Curaça, BA	2012	nd	nd	+
RS6		Red Globe	Brazil	Curaça, BA	2012	nd	nd	+
RS8		Red Globe	Brazil	Curaça, BA	2012	nd	nd	+
RS10		Red Globe	Brazil	Curaça, BA	2012	nd	nd	+
RS11		Red Globe	Brazil	Curaça, BA	2012	nd	+	+
XCV005		Red Globe	Brazil	Petrolina, PE	2008	+	+	+
XCV008		Red Globe	Brazil	Petrolina, PE	2008	+	+	+
XCV009		Red Globe	Brazil	Petrolina, PE	2008	+	+	+
XCV013		Sugraone	Brazil	Petrolina, PE	2009	+	–	+
XCV015		Sugraone	Brazil	Petrolina, PE	2009	+	+	+
XCV021		Thompson Seedless	Brazil	Petrolina, PE	2009	+	+	+
XCV026		Thompson Seedless	Brazil	Petrolina, PE	2009	+	+	+
XCV028		Sugraone	Brazil	Petrolina, PE	2009	+	+	+
XCV033		Sugraone	Brazil	Petrolina, PE	2009	+	–	+
XCV034		Thompson Seedless	Brazil	Petrolina, PE	2009	+	+	+
XCV039		Sugraone	Brazil	Petrolina, PE	2009	+	–	+
XCV040		Italia	Brazil	Petrolina, PE	2009	+	–	+
XCV044		Thompson Seedless	Brazil	Juazeiro, BA	2009	+	–	+
XCV045		Crimson	Brazil	Juazeiro, BA	2009	+	+	+
XCV047		Italia	Brazil	Juazeiro, BA	2009	+	–	+
XCV050		na	Brazil	Petrolina, PE	2009	+	–	+
XCV052		Sugraone	Brazil	Casa Nova, BA	2009	+	+	+
XCV054		Sugraone	Brazil	Casa Nova, BA	2009	+	+	+
XCV056		Sugraone	Brazil	Casa Nova, BA	2009	+	+	+
XCV065		Italia	Brazil	Casa Nova, BA	2009	+	+	+
XCV068		Red Globe	Brazil	Casa Nova, BA	2009	+	+	+
XCV070		Benitaka	Brazil	Casa Nova, BA	2009	+	+	+
XCV071		Sugraone	Brazil	Petrolina, PE	2009	+	–	+
XCV076		Sugraone	Brazil	Petrolina, PE	2009	+	+	+
XCV079		Red Globe	Brazil	Petrolina, PE	2009	+	+	+
XCV080		Sugraone	Brazil	Petrolina, PE	2009	+	+	+
XCV081		Thompson Seedless	Brazil	Casa Nova, BA	2009	+	+	+
XCV090		Thompson Seedless	Brazil	Juazeiro, BA	2009	+	+	+
XCV091		Thompson Seedless	Brazil	Juazeiro, BA	2009	+	+	+
XCV112		Red Globe	Brazil	Petrolina, PE	2009	+	–	+
XCV114		Thompson Seedless	Brazil	Petrolina, PE	2009	+	+	+
XCV116		Thompson Seedless	Brazil	Petrolina, PE	2009	+	+	+
XCV117		Red Globe	Brazil	Petrolina, PE	2009	+	+	+
XCV119		Thompson Seedless	Brazil	Petrolina, PE	2009	+	+	+
XCV124		Red Globe	Brazil	Petrolina, PE	2009	+	+	+
XCV129		Sugraone	Brazil	Juazeiro, BA	2010	+	+	+
XCV133		Sugraone	Brazil	Petrolina, PE	2010	+	+	+
XCV137		Thompson Seedless	Brazil	Casa Nova, BA	2010	+	+	+
XCV143		Red Globe	Brazil	Petrolina, PE	2010	+	–	+
XCV153		Red Globe	Brazil	Casa Nova, BA	2010	+	+	+
XCV154		Red Globe	Brazil	Casa Nova, BA	2010	+	+	+
XCV171		Sugra18	Brazil	Petrolina, PE	2011	+	+	+
XCV176		Red Globe	Brazil	Juazeiro, BA	2011	+	+	+
XCV178		Sugraone	Brazil	Casa Nova, BA	2011	+	+	+
XCV179		Benitaka	Brazil	Casa Nova, BA	2011	+	+	+
XCV181		Red Globe	Brazil	Juazeiro, BA	2011	+	+	+
XCV191		Thompson Seedless	Brazil	Juazeiro, BA	2011	+	+	+
XCV192		Thompson Seedless	Brazil	Juazeiro, BA	2011	+	+	+
XCV200		Red Globe	Brazil	Juazeiro, BA	2011	+	+	+
XCV201		Thompson Seedless	Brazil	Petrolina, PE	2011	+	+	+
XCV202		Sugraone	Brazil	Petrolina, PE	2011	+	+	+
XCV203		Thompson Seedless	Brazil	Petrolina, PE	2011	+	+	+
XCV204		Sugraone	Brazil	Petrolina, PE	2011	+	+	+
XCV207		Thompson Seedless	Brazil	Petrolina, PE	2011	+	+	+
XCV208		Sugraone	Brazil	Petrolina, PE	2011	+	+	+
XCV210		Sugraone	Brazil	Petrolina, PE	2011	+	+	+
Am-1		*Amaranthus* sp.	Brazil	Petrolina, PE	2012	+	+	+
Am-2		*Amaranthus* sp.	Brazil	Petrolina, PE	2012	nd	+	+
Am-3		*Amaranthus* sp.	Brazil	Petrolina, PE	2012	nd	+	+


*^a^States in Brazil: PE, Pernambuco; BA, Bahia; PI, Piauí; PR, Paraná; RR, Roraima; ^*b*^PCR with specific primers targeting a 240 bp-sequence of the *hrcN* gene (Trindade et al., 2007); ^*c*^na, information not available; ^*d*^not determined; ^*e*^year added to NCPPB.*

All strains were recovered on LPGA medium (yeast extract 7 g liter^-1^; peptone 7 g liter^-1^; glucose 7 g liter^-1^; agar 15 g liter^-1^, pH 7.2) and transferred to 10% TSA medium (1.7 g liter^-1^ tryptone, 0.3 g liter^-1^ soybean peptone, 0.25 g liter^-1^ glucose, 0.5 g liter^-1^ NaCl, 0.5 g liter^-1^ K_2_HPO_4_, and 15 g liter^-1^ agar). Cells were grown at 28°C for 24 h. For long term storage strains were preserved and frozen in 40% glycerol at -80°C. DNA was obtained from 1 × 10^7^ CFU ml^-1^ bacterial cell suspensions with a heating step at 94°C for 10 min before the amplification program.

### Multilocus Sequencing Analysis (MLSA)

Two strains from India (CFBP 7660 and CFBP 7691) and a subcollection of 26 strains from the 105-strain collection from Brazil were selected to represent the diversity in terms of year, host, and geographical origin. PCR amplifications of portions of seven housekeeping genes [*atpD*: ATP synthase- beta chain, *dnaK*: encoding the 70-kDa heat shock protein, *efp*: elongation factor P, *fyuA* coding a transmembrane protein (Ton-B dependent transporter), *glnA*: glutamine synthetase I, *gyrB*: DNA gyrase subunit B, and *rpoD*: RNA polymerase sigma 70 factor] were carried out with the primers designed by Mhedbi-Hajri et al. (2013), except for *gyrB* from which a 904-bp portion was amplified with the forward primer XgyrB1F (ACGAGTACAACCCGGACAA) and the reverse primer XgyrB1R (CCCATCARGGTGCTGAAGAT) (Young et al., 2008).

PCR amplifications were performed in a 25 μl-reaction containing 1X Go Taq Buffer (Promega), 200 μM dNTPs, 0.5 μM of each primer, 0.375 U of Go Taq Polymerase, and 5 μl of boiled bacterial cell suspension. Amplification program was carried out in a PE 9600 thermocycler (Applied Biosystems) with an initial denaturation at 94°C for 5 min, 35 cycles of denaturation at 94°C for 30 s, annealing at 60°C for 30 s (or 62°C for *efp*), extension for 1 min at 72°C, and a final extension at 72°C for 7 min. Quality and yield of PCR products were checked by loading 5 μl of the reaction in 1% agarose gels in 1 x Tris acetate EDTA (TAE) followed by staining with ethidium bromide. PCR products (20 μl) from each strain/gene combination were sequenced with reverse and forward primers at Genoscreen (Lille, France). Sequences obtained from forward and reverse primers were assembled and edited using GENEIOUS Pro 4.8.5 (Biomatters, New Zealand). Consensus sequences were generated, and codon-based multiple alignments were obtained using CLUSTALW (Thompson et al., 1994) application in BioEDIT (Hall, 1999) with default parameters. Initial phylogenetic analyses were performed on individual *rpoD* and *gyrB* sequences for comparisons with sequences from *Xanthomonas* (type and pathotype strains) from the CFBP/PhyloSearch tool database^1^ using the Neighbor Joining (NJ) method available in MEGA 5.05 (Tamura et al., 2007). As the concatenated data sets were identical for all 27 strains tested, we selected the sequences from the pathotype strain (CFBP 7660) for comparisons with DNA sequences of 131 strains of *X. axonopodis* representing 21 pathovars (Mhedbi-Hajri et al., 2013) from all six Rademaker’s genetic groups (Rademaker et al., 2005). Phylogenetic analysis was performed for each gene individually and on the concatenated data set. Concatenated alignments of the seven-genes sequences displayed in alphabetic order were generated in GENEIOUS to a final sequence of 4,759 bp (1–738 for *atpD*, 739–1485 for *dnaK*, 1486–1832 for *efp*, 1833–2473 for *fyuA*, 2474–3352 for *glnA*, 3353–4054 for *gyrB*, and 4055-4759 for *rpoD*). Separate and concatenated trees were constructed by NJ and maximum-likelihood (ML) reconstruction methods. For the latter, the model of nucleotide substitution was estimated with hierarchical likelihood ratio test (hLRT) and the Akaike Information Criterion (AICc) to select the best model from 56 candidate models, using Modeltest 3.7 in PAUP (Swofford, 2002). Phylogenetic trees were obtained by the PhyML method and *Xanthomonas campestris* pv. *campestris* strain CFBP5241 (ATCC 33913) was used to root the tree as it is more distantly related from the other xanthomonads (*X.* *citri* and related species). The SH test (Shimodaira and Hasegawa, 1999) from the DNAml program in PHYLIP (Felsenstein, 1989) was performed to test whether the ML tree topology based on each separate gene fell within the same confidence limits. For both NJ and ML trees, bootstrap analyses were performed with 1,000 replications and the trees were generated with MEGA 5.05.

### HR Induction and Pathogenicity Tests on *Vitis vinifera*

Upon isolation from plant material, strains were tested for induction of HR on tomato (*Solanum lycopersicum* “Santa Clara”) by leaf infiltration of a 1 × 10^9^ CFU ml^-1^ bacterial suspension in sterile distilled water. Pathogenicity of isolated strains was confirmed following infiltration of *V. vinifera* cv. Red Globe leaves with a bacterial suspension at 1 × 10^8^ CFU ml^-1^. Bacterial suspension (100 μL^-1^) was infiltrated into four points of the abaxial surface of the leaves with the aid of a hypodermic syringe without needle. These qualitative tests were conducted with two replicates per isolate. Inoculated plants were maintained in a greenhouse at 28°C and the pathogen was reisolated from typical lesions 7–10 days after inoculation.

The three pathotype strains (CFBP 7657, 7658, and 7659) were tested on 90- day-old *V. vinifera* plants cv. Sauvignon under controlled conditions (28°C, 98% RH, photoperiod of 16 h). Two methods of inoculation were employed: leaf infiltration (two spots per leaf) with 200 μl of a 1 × 10^7^ CFU ml^-1^suspension and deposition of 25 μl of a 1 × 10^8^ CFU ml^-1^suspension on the stems at three points after wounding with a needle. Two plants and six leaves per plant were inoculated with each strain. Plants were kept at 100% humidity for 48 h and were evaluated for symptom development until 35 days after inoculation. Three additional plants each were inoculated with pathovar *viticola* strains from India, CFBP 7660 and CFBP 7694, and one Brazilian strain (CFBP 7764) as positive controls. One plant was inoculated with water and kept as a negative control. All inoculation tests were carried out following quarantine procedures at IRHS, France. The assay was evaluated qualitatively, by scoring presence or absence of necrotic symptoms during 35 days. Isolations from inoculated leaves and stems were attempted 35 days after inoculation and colony growth was recorded after 48–72 h on 100% TSA medium.

### Selection of VNTR Loci for MLVA

Bacterial suspensions of each strain were prepared at 1 × 10^7^ CFU ml^-1^ and were boiled at 95°C for 10 min before PCR. Aliquots of boiled cells were kept at -20°C. Primers for amplification of 14 VNTR loci from *X. citri* pv. *citri* (Bui Thi Ngoc et al., 2009) were tested with five strains of pathovar *viticola* and strain 306 of pathovar *citri*. Reaction mix contained 1X GoTaq Flexi buffer (Promega), 1.5 or 3.0 mM MgCl_2_, 62.5 μM each dNTP, 0.125 μM of each primer, 0.25 U of GoTaq Flexi DNA polymerase and 1 μl of bacterial cells suspension. Conditions for amplification were as follows: 95°C for 5 min followed by 32 cycles of 95°C for 30 s; 60, 64 or 68°C, depending on the primer set, for 30 s and 72°C for 30 s and a final extension at 72°C for 10 min. PCR products were separated on 2.5% agarose gels in 1X Tris acetate EDTA buffer (TAE) and visualized after ethidium-bromide staining. When poor amplification occurred, PCR was optimized by testing different MgCl_2_ concentrations (1.5 and 3.0 mM) and annealing temperatures (60, 64, 68°C). VNTR loci were selected based on reproducibility (amplicons of same size produced in different PCR runs) and polymorphism detection among pathovar *viticola* strains, verified by gel electrophoresis.

### VNTR Genotyping

Eight VNTR loci were selected and the forward primers were tagged with one of four fluorescent dyes, 6-FAM, VIC, NED or PET (Eurofins MWG/Operon). Primer pairs were combined in four duplexes, with the respective annealing temperature, as follows: XL-1 FAM and XL-4 VIC, 64°C; XL-13 NED and XL-15 PET, 60°C; XL-3 FAM and XL-5 PET, 64°C; XL-6 NED and XL-8 VIC, 68°C.

PCR products (1 μl of products marked with 6-FAM, VIC or NED; and 2 μl of products marked with PET) were diluted in ultrapure water to a final volume of 32 μl. An aliquot of 2.4 μl was mixed with 0.15 μl of the GeneScan^TM^ 500 LIZ^TM^ size standard (Applied Biosystems) and 9.35 μl formamide in a 96-well tray, followed by denaturation at 94°C for 10 min in a thermocycler. Capillary electrophoresis was conducted in the ABI3130 sequencer using the GeneMapper application. Chromatograms were visualized with PeakScanner^TM^ software v. 1.0 (Applied Biosystems). Fragment sizes were estimated and converted into copy number for each VNTR. To confirm the copy number for each VNTR locus, PCR products of strains CFBP 7660 and CFBP 7764 were sequenced at Genoscreen. Sequences were edited with GENEIOUS and the search tools were used to detect the tandem repeat sequences.

### Stability Test

In order to test the stability of the pathovar *viticola* VNTR types after successive culture transfers, four distinct strains were tested: three pathovar *viticola* strains (CFBP 7660, 7764, and 5869) and *X. axonopodis* pv. *citri* strain 306. A starter bacterial cell suspension (1 × 10^8^ CFU ml^-1^) was prepared and 50 μl were transferred to 5 ml 10% TS liquid media. After 24 h- growth at 28°C, a new aliquot of 50 μl was transferred to a new tube. This procedure was repeated every 24 h for 4 days. At each day, a bacterial suspension of 1 × 10^7^ CFU ml^-1^ was prepared from each culture and tested for the eight VNTR makers. Serial dilutions and colony counts were performed on 10% TSA after 48 h to assess the number of generations from the starter culture.

### Analyzing VNTR Data

The VNTR data obtained from 107 strains of pathovar *viticola* were analyzed with BioNumerics (version 6.5, Applied Maths, Sint-Martens-Latem, Belgium). The copy numbers for each VNTR were used as character data and submitted to cluster analysis. A minimum spanning tree (MST) was generated. This tool creates a tree that connects all strains in such way that the summed distance of all branches is minimized. Clonal complexes were designed using BioNumerics. The Bayesian clustering approach was used to infer population structure and assign individuals to groups characterized by distinct allele frequencies (Pritchard et al., 2000). It was implemented in the software structure 2.3.4. The method estimates a probability of ancestry for each individual from each of the groups. Individuals are assigned to one cluster or jointly to two or more clusters if their genotypes indicate that they were admixed. Twenty independent runs of structure were performed by setting the number of subpopulations or groups (K) from 1 to 10, with 10,000 burn-in replicates and a run length of 20,000 replicates to decide which value of K best fits the data (Evanno et al., 2005). Clustering of isolates of pathovar *viticola* was evaluated for the inferred number of groups. Structure was run using the admixture model without prior population information, which assumes correlated allele frequencies for our MLVA data. The founder genotype, which is the one from which most single locus variants (SLV) arose (Feil et al., 2004; Spratt et al., 2004) was identified using eBURST v3^2^. The discriminatory power of MLVA was calculated using an online tool^3^.

### CFBP 7764 Genome Sequencing and *in silico* Design of New VNTRs

The genome of *Xanthomonas citri* pv. *viticola* strain CFBP 7764 was sequenced using the Illumina HiSeq 2000 platform (Genoscreen, France). The genomic sequence of another *X. citri* pv. *viticola* strain, LMG 965, already published (GCA_000723725.1) (Midha and Patil, 2014) was used for comparison. Annotation of both genomes was performed using EuGene-PP (Sallet et al., 2014). The genome sequences were mined to search CDSs encoding functions of interest for xanthomonads. A set of almost 1800 CDSs identified mostly in xanthomonads, but also in various pathogenic bacterial genera (*Xanthomonas, Pseudomonas, Ralstonia, Erwinia, Escherichia, Salmonella*) was used to screen for homologs of these proteins using tBLASTN (identity higher than 80% on at least 80% of CDS length). Genes encoding proteins involved in chemotaxis, motility, lipopolysaccharide and exopolysaccharide biosynthesis, TonB-dependent transporters (TBDTs), two-component systems (TCSs), the different secretion systems (T1SS, T2SS, T3SS, T4SS, T6SS) and their effectors, fibrillar and afibrillar adhesins, and insertion sequences (ISs) belonging to different families were included in this list. Furthermore, reciprocal tBLASTN (identity higher than 80% on at least 80% of CDS length) were performed between the 4,572 and 4,233 CDSs that were predicted in CFBP 7764 and LMG 965 genomes, respectively.

Tandem repeats finder web tool (Benson, 1999) was used to search Variable Number of Tandem Repeats (VNTRs) in the genome of CFBP 7764. Selected VNTRs have at least two copies with period size shorter than 100 nucleotides and a percentage of matches of at least 95% between the different copies. VNTRs were then checked on LMG 965 genome and only VNTRs that had a different number of copies within both genomes were selected. Primers conserved in both strains were designed in the 500 bp-flanking regions using Primer3 web site (Untergasser et al., 2012) in order to amplify DNA fragments with a final size between 100 and 350 bp to be compatible with the use of the ABI3130 capillary electrophoresis sequencer.

### Nucleotide Sequence Accession Numbers

The GenBank accession numbers for the partial sequences of the grapevine strain CFBP 7764 and the *Amaranthus* strain Am-1, used in this study are, respectively: for *atpD* MH171285 and MH171286*;* for *dnaK* MH171287 and MH171288*;* for *efp* MH171289 and MH171290*;* for *fyuA* MH171291 and MH171292*;* for *glnA* MH171293 and MH171294*;* for *gyrB* MH171295 and MH171296*;* and for *rpoD* MH171297 and MH171298.

### Accession Number

The whole-genome shotgun sequence of *Xanthomonas citri* pv. *viticola* strain CFBP 7764 has been deposited in GenBank under accession no. PPHE00000000.

## Results

### *Xanthomonas* Strains Causing Grapevine Bacterial Canker in Brazil Belong to *X.* *citri* pv. *viticola* and Are Monomorphic Based on MLSA

**FIGURE 1:**
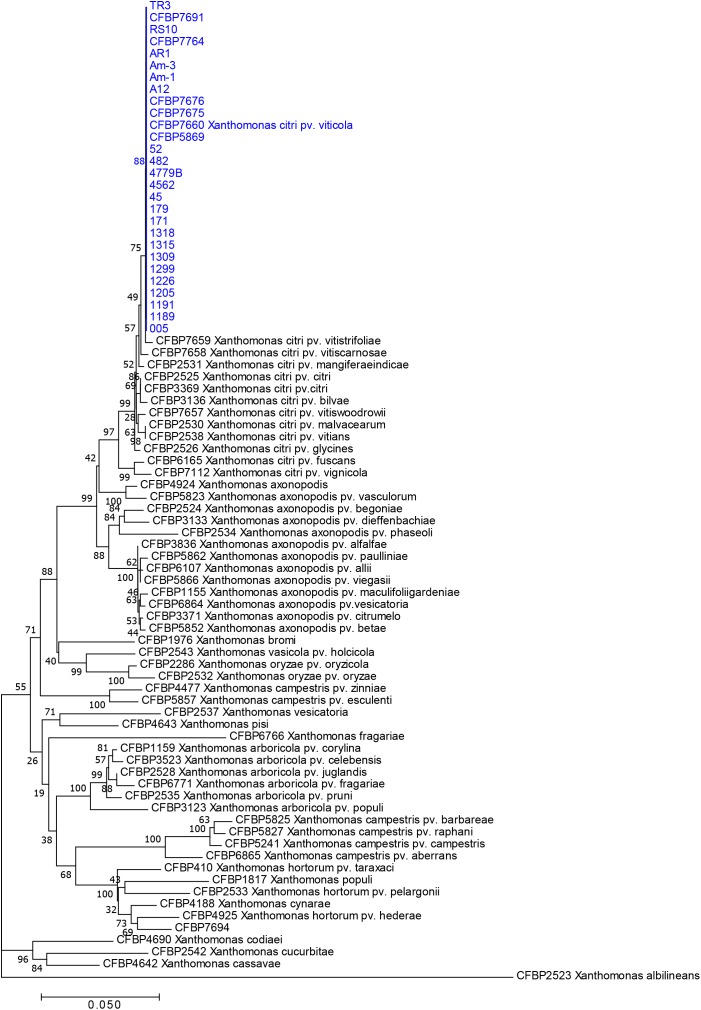
Neighbor-joining tree based on concatenated partial sequences of *gyrB* and *rpoD* of 28 strains of pathovar *viticola* and 15 type strains of most *Xanthomonas* species. Bootstrap values (1,000 replicates) are shown at each node. The 28 strains include 24 strains isolated in Brazil from bacterial canker-infected grapevines, two (Am-1 and Am-3) from *Amaranthus* plants grown in the vicinity of grapevines, and two strains from India (CFBP 7660 and CFPB 7691). CFBP 7660 is the pathotype strain of *X. citri* pv. *viticola*.

Neighbor Joining tree-based phylogeny determined from *rpoD* and *gyrB* concatenated sequences alignments showed the relatedness among types and pathotypes of 15 *Xanthomonas* species and 27 strains of pathovar *viticola* and strains of the three other pathovars affecting plants in the Vitaceae family (Figure 1). All pathovar *viticola* strains (Table 1) had identical sequences for both gene sequences, including the Brazilian and Indian strains and two strains from *Amaranthus* sp. These strains, as well as those from the related pathovars affecting Vitaceae species from India, were assigned to the newly described *X. citri* species that encompasses the previously described 9.5 and 9.6 groups. The pathovar *viticola* strains were all distinct from these other pathovars. Based on housekeeping gene sequences, the closest relative to pathovar *viticola* is pathovar *vitistrifoliae*.

**FIGURE 2:**
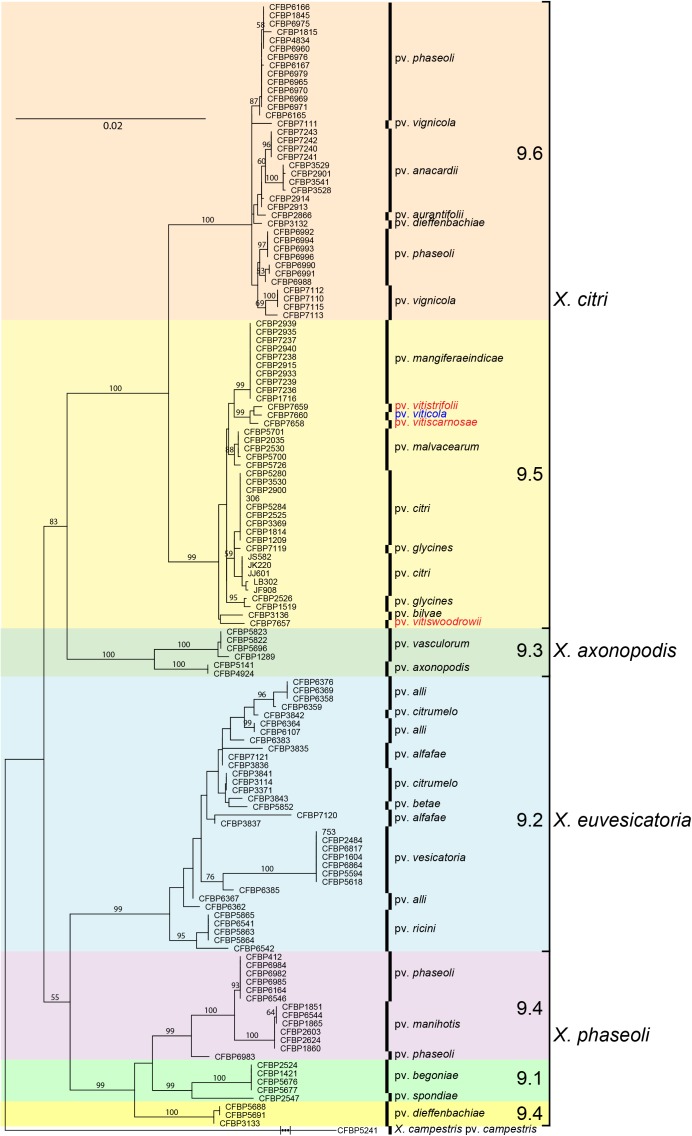
Maximum likelihood tree of 131 *Xanthomonas* strains based on the 4,759 bp concatenated sequences of *atpD*, *dnaK*, *efp*, *fyuA*, *glnA*, *gyrB*, and *rpoD*. Tree was constructed with PhyML and the bootstrap values higher than 50 (1,000 replicates) are shown at each node. Sequences of the pathotype strain (CFBP 7660) of *X. citri* pv. *viticola* were compared to sequences of 131 strains formerly assigned to *X. axonopodis* representing 21 pathovars (Mhedbi-Hajri et al., 2013) and all six Rademaker’s genetic groups 9.1–9.6 (Rademaker et al., 2005). Correspondence between Rademaker’s groups and the four *Xanthomonas* species (according to Constantin et al., 2016) are indicated. *Xanthomonas campestris* pv. *campestris* strain CFBP 5241 (ATCC 33913) was included as outgroup.

Sequences of all seven genes were identical for all strains collected from grapevine and from *Amaranthus*. Therefore, only one sequence type was used for comparisons with 131 gene sequences from *X. axonopodis* pathovars from Mhedbi-Hajri et al. (2013) and sequences from the three Vitaceae-associated pathotypes. ML (Figure 2) and NJ trees were constructed based on the 4,759 bp concatenated sequences of the seven genes. Both trees showed congruent assignments for most pathovars according to Rademaker’s genetic groups 9.1–9.6, except for pathovars alfalfae and allii from the 9.2 group. ML trees showed higher bootstrap values compared to the NJ trees. Both methods assigned the pathovar *viticola* and the other related pathovars to the 9.5 clade. The SH test performed on the ML trees showed that for five genes the concatenated tree topology was congruent with each individual gene tree, except for *gln*A and *rpo*D (Supplementary Table S1). For these two genes, different positions of pathovars in the 9.1 and 9.2 groups were evident, suggesting occurrence of recombination events.

**FIGURE 3:**
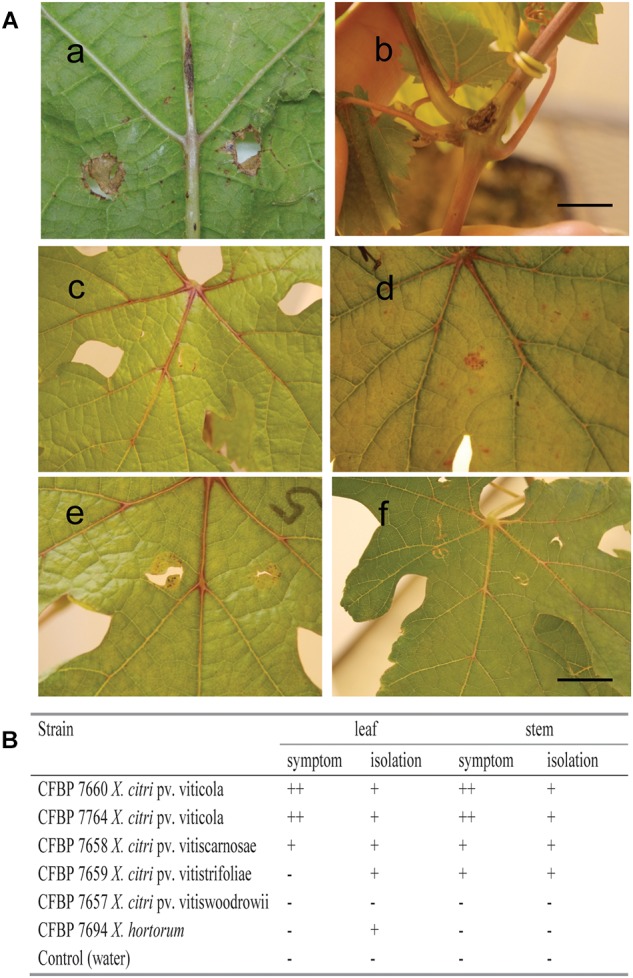
Pathogenicity test on *Vitis vinifera* cultivar Sauvignon carried out by leaf and stem inoculations with *Xanthomonas* strains. **(A)** Symptoms 35 days after inoculation of: CFBP 7764 on leaf and stem **(a,b)**; CFBP 7657 **(c)**, CFBP 7658 **(d)**, CFBP 7659 **(e)** and negative control at 21 days after infiltration **(f)**. **(B)** Symptom development was recorded as (+) necrosis at the point of infiltration; (++) necrosis at the point of infiltration followed by multiple necrotic spots on the leaves and leaf veins, or development of canker-like lesions on the stems; strain CFBP 7694 was received as *X. campestris* pv. *viticola*, but is related to *X. hortorum* according to *gyrB* and *rpoD* sequencing (as shown in Figure 1). Scale bar = 1.0 cm.

Based on MLSA the four pathovars affecting distinct species in the family Vitaceae were distinct from each other, while still belonging to *X. citri*, more precisely to the 9.5 Rademaker’s group (Figure 2). Pathovars *vitistrifoliae* and *viticola* fell into one clade supported by high bootstrap values. On the other hand, pathovar *vitiswoodrowii* fell into a different clade, closest to pathovar bilvae. These three strains from India are reported as non-pathogenic on *V. vinifera*. However, due to the differences in their phylogenetic positions (Figure 2) we tested them for pathogenicity on *V. vinifera*, cv. Sauvignon. These pathogenicity tests confirmed the non-host status of *V.* *vinifera* only for pathovar *vitiswoodrowii*. Symptoms developed on stems of plants inoculated with pathovars *vitiscarnosae* and *vitistrifoliae*. For pathovar *vitiscarnosae*, necrotic spots at the point of infiltration also developed on leaves (Figure 3A). After 35 days, the bacterium was isolated from both leaves and stems of all plants, but isolations were unsuccessful from plants inoculated with pathovar *vitiswoodrowii*. While symptoms incited by these two pathovars were mild and did not progress beyond the point of infiltration, the plants inoculated with pathovar *viticola* strains CFBP 7660 and 7764 showed more severe symptoms. Besides leaf perforation, several spots appeared on the leaf veins and in interveinal areas of the leaf that gradually enlarged becoming necrotic. Grape leaves inoculated with pathovar *vitistrifoliae* did not show any symptoms, but the isolation was positive, yielding pure colonies of the bacterium (Figure 3B).

It should be noticed that strain CFBP 7694 (NCPPB 3642), received *as Xanthomonas campestris* pv. *viticola*, was assigned to the *X. hortorum* clade, and for this reason it was not included in the MLVA study. In contrast to all pathovar *viticola* strains, which are non-pigmented, CFBP 7694 was a yellow-pigmented strain isolated in India and added to NCPPB in 1990. Due to its atypical characteristics, this strain was also tested for pathogenicity on grapevine plants. The pathogenicity test showed that it is not pathogenic on this host. This strain was identified as *X. hortorum*, according to *gyrB* and *rpoD* sequence analysis (Figure 1). No symptoms were observed on leaves or stems 35 days after inoculation. However, bacterial colonies were isolated from inoculated leaves, suggesting that this strain can survive in grape leaves (Figure 3B).

### VNTR Markers From *X.* *citri* pv. *citri* Have Sufficient Resolution to Detect Diversity in Pathovar *viticola*

Out of 14 VNTR loci from *X. citri* pv. *citri* (Bui Thi Ngoc et al., 2009), 13 were PCR-amplified from DNA of pathovar *viticola* strains. Primers for marker XL-2 did not produce any visible fragments on agarose gels. Using a subset of five pathovar *viticola* strains and strain 306 of *X. axonopodis* pv. *citri*, polymorphism was observed with eight markers (Table 2). The stability of these markers was checked *in vitro* after 32 generations for four strains, three pathovar *viticola* strains (CFBP 7660, 7764 and 5869) and *X. axonopodis* pv. *citri* strain 306. No variation in fragment sizes was observed throughout the experiment. Hence, all eight markers remained stable after 32 generations of these four strains.

**Table 2:** Number of alleles, range of repeat numbers, strain frequency for each dominant allele and allelic diversity for the eight VNTR loci tested on 107 pathovar *viticola* strains.

Locus	Number of alleles	Range of repeat numbers	Dominant allele and strain frequency (%)	Simpson’s diversity index
XL 1	12	5–19	13 (29.9)	80.5
XL 4	11	5–16	11 (33.3)	80.8
XL 13	5	4–10	7 (85.0)	26.6
XL 15	8	3–11	10 (77.6)	39.0
XL 3	12	6–18	11 (27.1)	86.7
XL 8	5	4–13	5 (88.8)	19.3
XL 6	12	8–29	24 (30.8)	80.3
XL 5	7	6–13	7 (52.3)	64.1


### MLVA Typing Revealed Diversity in the Pathogen Population From Brazil

**FIGURE 4:**
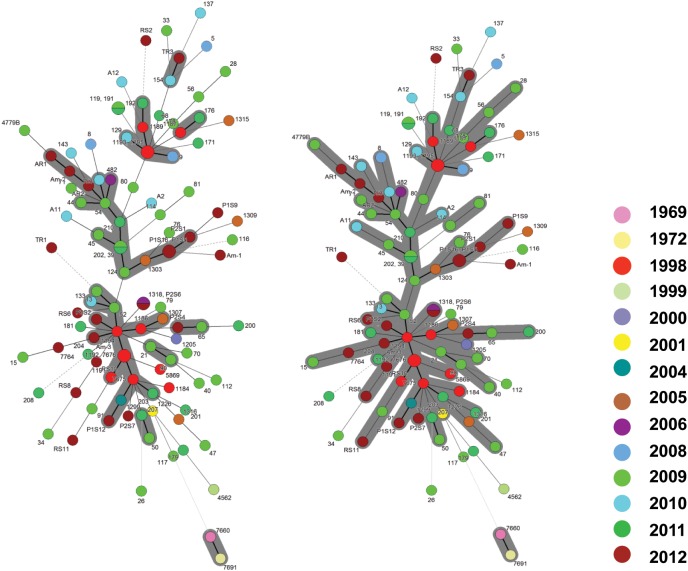
Minimum spanning trees of 107 *Xanthomonas citri* pv. *viticola* strains, comprising 105 Brazilian strains and two strains from India (CFBP 7660 and 7691), based on MLVA with 8 VNTR markers. The circles represent a MLVA type. The types that are connected by a thick solid line differed by 1 VNTR locus; MLVA types connected by thin solid lines differed by 2–3 VNTR loci, and the types that differed by 4 or more loci are connected by dashed and dotted lines. **(A)** The gray zone represents clonal complexes comprising MLVA types that differ from one another by one locus, **(B)** the gray zone groups types that differ by one or two loci.

**FIGURE 5:**
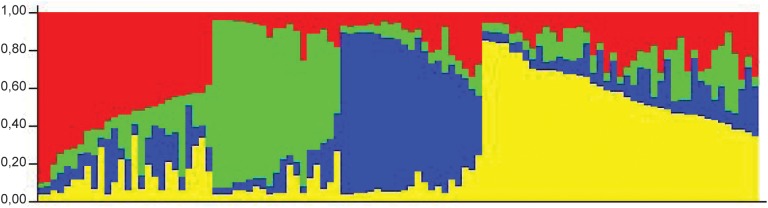
STRUCTURE outputs for a test panel of 107 strains of *Xanthomonas citri* pv. *viticola*. Best K, the true value for number of clusters, was selected using the Evanno method (Evanno et al., 2005). Colors represent groups identifiable by Bayesian clustering.

**FIGURE 6:**
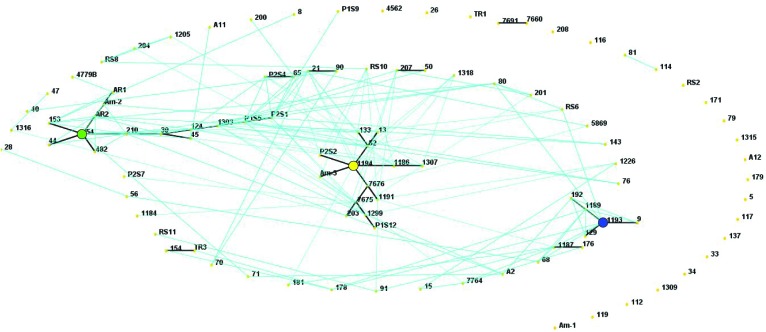
E-BURST network based on eight VNTR markers showing single locus variants (thick dark lines) and double locus variants (light blue lines) in a collection of 107 *Xanthomonas* *citri* pv. *viticola* strains, comprising 105 Brazilian strains and two strains from India (CFBP 7660 and 7691). The predicted founders of each clonal complex (strains 1193, 1194 and 54) are indicated with colored solid circles. The colors correspond to their groups in the Structure analysis (Figure 5).

In a collection of 107 pathovar *viticola* strains, the number of alleles ranged from 5 to 12 and the copy numbers of the repeat sequences ranged from 3 to 29. Four VNTR loci were the most diverse (diversity indexes over 80%): XL-1, -4, -3, and -6. The other four revealed less diversity in the collection. For example, for XL-8, 95 strains (88.8%) presented the same allele (Table 2). A total of 101 haplotypes were detected, but none of them was overrepresented in this set of strains. The discriminatory power of the MLVA was calculated and it showed a level of discrimination of 0.9563 for 107 typed strains. A MST based on repeat copy numbers shows the relationships among 107 strains in relation to the year of isolation and a subdivision in several clusters (Figure 4). The VNTR markers clearly separated the two Indian strains from the Brazilian strains. These strains from India, isolated in 1969 and 1972, had unique alleles at three loci (XL-4, -8, and -5) and differ from each other by one mismatch at locus XL-1. Three larger clonal complexes composed by strains that differed by only one VNTR were detected in the Brazilian set of strains. Two of these complexes contained older strains, which were isolated in 1998, the year of the first disease outbreak in Brazil. Bayesian clustering was performed in Structure supporting four groups (*K* = 4). Analysis of these groups revealed one population with greater admixture containing the Indian strains, two groups containing isolates from 1998 to 2012 and one group with isolates from 2006 to 2012 with overlapping (Figure 5). The E-burst algorithm identified also three clusters of related genotypes, and several singletons (Figure 6). The predicted founders for the three clusters are strains 1193, 1194 and 54. Strains 1193 and 1194 were both isolated from Red Globe vines in 1998 in Petrolina, state of Pernambuco, but from two different vineyards. However, when grouping the strains that shared identical alleles at 6 or 7 loci, one single large clonal complex appeared (Figure 4B, 6). The predicted founder of this larger complex was strain 1194 from which the larger number of single and double locus variants emerged.

The two strains (4779B and 482) that were more geographically distant (i.e., detected in the states of Parana, south of Brazil, and Roraima, northwest of Brazil, respectively) had unique alleles at loci XL- 6 and XL- 1, respectively. Several strains appear as singletons not belonging to the three major clonal complexes (eBURST and MST), for example, strain 26 (Figure 4A). This strain has one unique allele at locus XL-15 and it is also unique as far as to its collection site. It was collected in 2009 in the same municipality (Petrolina) as most others, but it is the only strain in the collection from one specific grape-producing area.

Regarding the host of isolation, the three strains from *Amaranthus* (Am-1, Am-2, and Am-3) had three distinct MLVA profiles and those were not identical to any of the strains collected from grapevines in the same area, in the same year (P1S5, P1S6, P1S9, P1S12, and P1S16). Strain Am-1, for example, had different copy numbers for 3 VNTRs compared to strains P1S5 and P1S16, that share the same MLVA type. Only one VNTR locus (XL 13) was monomorphic among the three Amaranthus and the five grape strains collected in the same area and year. Interestingly strain Am-3 and the founder genotype of one of the clonal complexes (strain 1194) were identical in 7 loci (Figure 4).

### CFBP 7764 Genome Features Are Typical of Plant Pathogenic Xanthomonads

Strain CFBP 7764 was chosen for whole genome sequencing analysis because it was isolated in Brazil with a time lapse of more than 40 years compared to the Indian pathotype strain. Shotgun sequencing yielded 8,390,830 100-bp paired-end reads with an insert size of 250 bp. A combination of Velvet (Zerbino and Birney, 2008), SOAPdenovo, and SOAP Gapcloser (Luo et al., 2012) yielded 76 contigs (N50, 592,828 bp), with the largest contig being 791,586 bp, for a total assembly size of 5,311,793 bp. Genomic sequence of this strain CFBP 7764 showed a typical *Xanthomonas* gene content (Alegria et al., 2005; Potnis et al., 2011). The genes encoding the main secretion systems described in Gram-negative bacteria were detected in the genome of strain CFBP 7764. Genes encoding at least two T1SSs and two more putative T1SSs were identified. Genes encoding proteins involved in Tat and Sec pathways, in two complete T2SSs (Xcs and Xps) and 77 putative T2-secreted cell wall degrading enzymes were predicted. The *hrp* cluster encoding the T3SS-Hrp2 family and 17 T3E-genes (*avrBs2, xopA, xopAE, xopAI, xopAQ, xopB, xopC2, xopE1, xopE3, xopK, xopL, xopN, xopP, xopQ, xopV, xopX, and xopZ1*), a T4SS gene cluster similar to the chromosomic cluster of Xac306 (Alegria et al., 2005), and a single T6SS cluster belonging to the group 3 (Potnis et al., 2011) were predicted. Strain CFBP 7764 is fully equipped with genes necessary to sense and move in its environment, to protect itself, and to acquire nutrients, through a complete flagellar system, at least 25 MCPs (methyl-accepting chemotaxis proteins), complete type I and type IV pili, several T5SS, including *fhaB, fhaC, shlB*, and *yapH*, xanthan biosynthesis and near seventy TBDTs. At least, almost 120 genes encoding TCSs could be involved in the detection and the response to environmental signals. Comparison of the genomic sequences of the two strains of *X. citri* pv. *viticola* did not show any differences in all these functions. The draft quality of the genome sequences did not allow an exhaustive analysis of IS content. It was however possible to observe some diversity between both strains. CFBB 7764 harbored partial sequences homologous to ISXac2 and ISXc8 from IS3 family that were not detected in LMG 965 sequence. Reciprocally, LMG 965 had sequences homologous to IS1477 from IS5 family that was not detected in CFBB 7764 (Supplementary Table S2).

As in strain LMG 965, the xanthomonadine biosynthesis gene cluster showed a truncated gene that can explain the white aspect of the colonies, in contrast to the yellow colonies of most species of the genus *Xanthomonas* (Midha and Patil, 2014). Comparison based on reciprocal tBLASTNs of the genomic sequences of CFBP 7764 and LMG 965 revealed 26 CDSs predicted in LMG 965 genome that had no orthologs in CFBP 7764, most of them were probably on a 16 Kb plasmid in LMG 965. Conversely, 233 CDSs predicted in CFBP 7764 genome had no orthologs in LMG 965 (Supplementary Tables S2, S3). These CDSs were distributed in several clusters, corresponding to almost 15 whole contigs of various sizes (between 64.4 and 0.8 Kb). Around 100 of these 233 CDSs had no orthologs in NCBI nr database. Most of the remaining CDSs had orthologs in plasmid sequences, such as plasmid pB07007 of *X. hortorum* strain B07-007, plasmid C of *X. citri* pv. fuscans strain 4834R, plasmid pICMP7383.2 of *X. gardneri*, and plasmid pLH3.1 of *X. euvesicatoria* pv. perforans strain LH3. Apart from numerous CDSs encoding proteins involved in conjugation, these putative plasmids carried CDSs encoding functions such as toxin-antitoxin, restriction and anti-restriction proteins, multidrug efflux systems and copper resistance genes (Supplementary Table S3). A *copLAB* gene cluster has hence been evidenced in CFBP 7764.

### Availability of Epidemiological Contrasted Genome Sequences to Design New VNTRs

The eight VNTRs used in this study were initially developed for *X. citri* pv. *citri* (Bui Thi Ngoc et al., 2009). These VNTRs were found within the two genomes of *X. citri* pv. *viticola* strains, being, however, slightly divergent (Supplementary Tables S4, S5). All VNTRs had a 7-nucleotide repeat motif and were distributed among six different contigs in both genomes. Genome mining showed that five of these eight VNTRs (XL3, XL 4, XL5, XL 8, and XL15) have different repeat numbers in these two strains isolated at a 43-year interval in different continents (Supplementary Table S4). Except for XL 5 and XL6, the numbers of repeats and number of loci with different copy numbers between the two strains were greater in the experiments (amplicon sequencing) than in the genome mining-based prediction. This was due to degenerated repeats that were not taken into account in the prediction using the Tandem Repeats Finder tool. Taking the opportunity of having these two genome sequences, we designed a set of 32 new VNTRs (Table 3). VNTRs were selected based on a repetition number higher than two, a length shorter than 100 bp, a high motif conservation within the VNTR (95%) and different repetition numbers between the two genome sequences. This VNTR scheme included repeats with motifs varying from three to 16 nucleotides and covering 11 different contigs, in particular five and nine VNTRS were designed in the two large contigs from CFBP 7764 (G102 and G103) that were not targeted with the *X. citri* pv. *citri* VNTR scheme, giving a wider representation of the entire genome sequence.

**Table 3:** VNTR scheme designed based on CFBP 7764 and LMG 965 genome sequences.

VNTR					CFBP7764		LMG 965	
Name	Repeat sequence	Period size	Left primer	Right primer	Copy number	Amplicon size	Copy number	Amplicon size
7764V1	GCTGtC	6	caattcagtcagggcgattt	tagaaaaacgtcgcgcatc	12.5	323	6.5	326
7764V2	TcGGGAA	7	aagacgttgacgccaaaaag	ttagcgagcaccgtaaggac	7.4	305	8.4	351
7764V3	GCAACGG	7	cctatcgacggtccgtttta	cccttcctcctcttccaact	4.6	268	5.4	275
7764V4	CATCGCCCAA	10	gacggtgtttcgggaatg	cggcacctatctggcatatc	2.9	300	1.8	290
7764V5	TCGGGAA	7	catcatggtagcggtggag	tggaaatcaacagcgacaac	6.4	308	5.3	301
7764V6	TgGGGAA	7	atacaggtgcccgaaggttt	aagcgcacatggcaataag	8.1	296	6	282
7764V7	CTTCTG	6	gtaaggaaagcgccctcac	gtgtgtgagcgtcagaaagg	6.3	311	4.8	302
7764V8	GAATCGG	7	caacaggccgagagatcatt	ccgtacactccggttctgag	5	297	7	311
7764V9	GCCCATCGCAT	11	gtcgtacggtgatgcaagtg	atggattttgctgctgtgtg	6.5	312	4.5	290
7764V10	GCCAAT	6	gggattcgggatttgctaac	gagctgagttgaccgtggag	4.8	300	5.8	306
7764V11	GCG	3	aacacctgacccttcgatca	cggatgcagcagatggac	12.3	291	11.3	288
7764V12	ATTCCCc	7	aatcgggaatggagaaaagc	cgccactacgccacctat	13.9	296	16.9	317
7764V13	GTGGCA	6	aggtcatcgtgccgtcttc	gtaaccccatcgcctacaag	4.3	293	5.3	299
7764V14	GTGTTG	6	gctgtgggatgtttgctttt	tccactcacaactcgacagc	11	301	9	289
7764V15	GAATCGG	7	gatggcgttcgaatacctg	ccaggatcaggaggctacag	6	315	5	308
7764V16	GGGCTGC	7	gttcggacatccaccgtatc	ggcggctagttctttgtcag	7.3	271	5.3	257
7764V17	CCCGAAT	7	agttgtacaaggcgcgctaa	ttgctgaagcagcaggatag	10.7	296	5.7	261
7764V18	TCGTGAA	7	ttgtcatcgtggaagtttcg	cggagagacgttgggtaaga	7.4	302	8.4	309
7764V19	ATTCCCG	7	ccggttatctggtcaacga	gcctggtcgttgatatagcc	4.9	300	5.9	307
7764V20	GCGAGAT	7	ggtgctgactggttgaaggt	gggtcactcgacatcggtat	3.6	315	2.4	308
7764V21	GGGAAGC	7	gcattgagggcggttagat	cagcacgttgtggttggat	6.1	294	7.1	301
7764V22	TGTAGA	6	ccttgcagtcgtccatacct	gagatcggtcggtggatg	6.3	336	8.3	348
7764V23	ACGCATC	7	gccgagtgaccgaaaacg	cagcagtcccaacacgaac	4	311	5	318
7764V24	AATCGGa	7	gtgcaatcggttgaaatgc	accttgccgctgtattacga	4.7	321	13.7	384
7764V25	GGCAGAA	7	tacagatcggtgtcgagcag	gtggtcagtcgcgctaaatc	4.7	313	2.6	299
7764V26	GCACCATCGCCACAAC	16	tacagacgtgggcggtgtat	gatgacatggaaacgcaaaa	5.2	297	6.2	312
7764V27	GCACCG	6	atcggctcggtgcgtatt	gctatcgcaaactggatcgt	8.5	298	6.5	286
7764V28	tTCCCGA	7	gctcaggaacgttgaagcat	atccgctcgatcatcgtc	8	291	10	305
7764V29	CGTCGATCCCCG	12	tgaatcaggtccacatgagc	caccacgtcgtactccactg	2.2	326	1.1	314
7764V30	CGTTGTG	7	gtgtcggtggacgtggat	ctctgtgcactgcggtaatg	8.4	301	13.4	336
7764V31	TTTCCGA	7	gcgcacaaacaaacaaaaag	gtcgcagctgttcaaggaat	13.3	266	9.3	238
7764V32	CCGAATC	7	gacgctgctagaatgacagc	tgagtcaggcggatcttctt	5.1	298	6.1	305


## Discussion

Although it was first described in 1972, the emergence of *X. citri* pv. *viticola* as a grapevine pathogen is relatively recent with outbreaks in India (1990) and Brazil (1998). Analysis of a Brazilian collection of strains showed that this pathovar lacks genetic diversity in seven housekeeping genes and confirms its status as a monophyletic pathovar of *X. citri* species. Further knowledge of the diversity of this pathogen was possible through a MLVA scheme with eight VNTR loci which allowed a better understanding of the genetic structure of the Brazilian strains.

Primers for amplification of VNTR loci in bacterial plant pathogens have been designed from draft or complete genome sequences (Arrieta-Ortiz et al., 2013; Cesbron et al., 2014; Cunty et al., 2015; Poulin et al., 2015) or from genomes of close relatives (Pruvost et al., 2011). VNTR markers developed from a specific pathovar genome can be successfully used for genotyping other pathovars belonging to the same species, as shown for *X. arboricola* pv. *pruni* and related pathovars (Cesbron et al., 2014). For pathovar *viticola*, the genome sequence of the reference strain was not available at the beginning of this study, consequently VNTR markers designed for the citrus canker pathogen *X. citri* pv. *citri* (*Xanthomonas* *axonopodis* pv. *citri*) were tested. Pathovar *citri* is phylogenetically related to pathovar *viticola* based on *gyr* B sequences (Parkinson et al., 2009) and sequences from other housekeeping genes (Gama et al., 2018; this study). A closer relationship between these two pathovars had been previously demonstrated by whole-cell fatty acid methyl esters (FAMEs) following a comprehensive study of 975 xanthomonads strains (Yang et al., 1993). In fact, diseases caused by both pathovars, *viticola* and *citri*, were first noted in India and recent whole genome comparisons confirm that the two pathovars are members of the same species but with different host specificity (Midha and Patil, 2014; Bansal et al., 2017).

Eight out of 14 VNTR markers described for pathovar *citri*, were polymorphic for pathovar *viticola*. Six out of these eight markers can also reveal polymorphism among strains from the pathovars *mangiferaeindicae* and *malvacearum* (Bui Thi Ngoc et al., 2009), which also belong to the rep-PCR group 9.5 (Rademaker et al., 2005; Mhedbi-Hajri et al., 2013). Both pathovars have been included in the newly described *X. citri* species (Constantin et al., 2016).

Compared to other methods for deciphering population structures and diversity, MLVA has much higher resolution, and can be applied to human pathogens that lack diversity in housekeeping genes, i.e., monomorphic (Achtman, 2008). Among plant pathogens examples of monomorphic pathogens are *Pseudomonas syringae* pv. *actinidae* biovar 3 (Cunty et al., 2015) and *X. citri* pv. *citri* (Pruvost et al., 2014; Leduc et al., 2015). In a similar way, MLSA approach lacks resolution to distinguish among strains of pathovar *viticola*. Strains from India and Brazil were identical in all seven genes. Four (*dnaK*, *fyuA*, *gyrB*, and *rpoD*) out of these seven loci were also used in the MLSA scheme proposed by Young et al. (2008) for species differentiation in *Xanthomonas*. Consequently, the VNTR markers were chosen to help us gain some insight and an overview of the genetic structure of pathovar *viticola* strains isolated in Brazil since the 1998 outbreak and to understand how these strains are linked to the Indian strains. The MLVA scheme with eight loci proved to be efficient tool for discriminating strains that had identical housekeeping genes sequences (Figure 4). Even though strains isolated in the same year, in the same location and from the same cultivar (many isolated from Red Globe vines) are overrepresented in the collection, MLVA had enough resolution to distinguish strains from the same area, strains from a weed host and grapevine, and to distinguish most Brazilian strains from the strains from India.

Grapevine bacterial canker is a disease with limited distribution around the globe. It was reported from India more than 40 years ago, but only in the last 20–25 years, it gained economic importance. Serious disease outbreaks occurred in India in the late 1980’s and were linked to increases in the area cultivated with the susceptible seedless cultivars (Chand and Kishun, 1990). The reported yield losses in severely infected vineyards were up to 60 or 80%. In 1998, the disease was first noted in Brazil affecting mostly seedless varieties. Currently, regarded as a quarantine pest in Brazil, control measures based on surveillance and eradication have been adopted (Naue et al., 2014). The detection of infected plants in other states and regions in the country (Halfeld-Vieira and Nechet, 2006; Rodrigues Neto et al., 2011) reveals pathogen spread by asymptomatic propagating material, which leads ultimately to eradication procedures. In the state of São Paulo, approximately 4,700 plants were destroyed due to a disease outbreak in 2009 (Rodrigues Neto et al., 2011).

A possible introduction event associated with propagating material originating from India has been hypothesized to explain the emergence of this disease in Brazil (Rodrigues Neto et al., 2011). This event should have taken place at least 3 years before the disease outbreak in 1998, since the first symptoms were observed on young vines up to 3 years of age. The lack of sequence variation in seven housekeeping genes among Brazilian and Indian strains shows that, globally, it is a monomorphic pathogen. A genetically monomorphic pathogen may arise from a strong reduction in the population size of the ancestors of the existing strains due to a recent bottleneck (Achtman, 2008). Housekeeping genes encodes essential metabolic enzymes for species survival, thus they may undergo strong purifying selection, as demonstrated for most phylotypes of the plant pathogen *Ralstonia solanacearum* (Castillo and Greenberg, 2007).

A panel of eight polymorphic VNTR markers derived from *X*. *citri* pv. *citri* was developed for *X*. *citri* pv. *viticola* and showed genetic diversity in a set of 105 strains from Brazil. The high discriminatory power of MLVA revealed patterns of genetic diversity nor detected by previous studies with rep-PCR (Trindade et al., 2005; Gama et al., 2018). MST and Structure analyses identified three congruent major genetic groups in the Brazilian collection. The epidemic-related strains from 1998 were separated in two groups while the two strains from India were clustered. A fourth group (red) detected by Structure (Figure 5) was not clearly understood as it groups the two strains from India with 23 strains from Brazil that were, mostly, not connected to the major MST clonal complexes and appear as singletons. Furthermore, admixture among populations was observed (Figure 5).

Some strains found in the same field, from the same grape cultivar and year of collection shared the same haplotype (P1S5 and P1S16; 1193 and 1195). However, same haplotypes were also shared by strains collected from neighboring states (CFBP 7676 and 1192; 191 and 119), which suggests dissemination by the planting or grafting of symptomless contaminated plant material. That would also explain the disease outbreaks in two more distant states in the country (Paraná, in the southeast and Roraima, in the north region).

Most Brazilian strains are members of three larger clonal complexes (Figure 4A). The predicted founder genotypes of two clonal complexes are strains 1194 and 1193 which were isolated in 1998 from Red Globe vines. These strains are not linked to the two Indian strains isolated in 1969 and in 1972. The fact that the Brazilian strains from 1998 belong to two distinct clonal complexes suggests that the 1998 outbreak of grapevine bacterial canker in Brazil probably occurred through one introduction event of two distinct grapevine planting materials contaminated with genetically distinct strains. The development of the irrigation projects in the São Francisco River valley in Brazil started in the 1970’s and the introduction and exchange of propagating material of different grape varieties occurred over time.

The lack of a more diverse and recent collection from India did not allow us to draw conclusions about the events that lead to the emergence of this pathogen in Brazil. We hypothesize that the 1998 outbreak-related strains from Brazil are probably epidemiologically linked to the strains that caused the severe disease outbreaks in India in the late 1980’s (Chand and Kishun, 1990) which were highly aggressive on seedless varieties, but not linked to the ancient strains (1969/1972) as shown by the results.

The environment where conditions are variable may favor the existence of more genetically diverse populations, from which new crop strains emerge, often as highly virulent clones (Goss et al., 2013). Alternative hosts harboring potential sources of inoculum may contribute to amplify the diversity observed in Brazil. In Brazil, xanthomonads-like bacteria have been isolated from several weeds growing in the vicinity of vineyards. Their pathogenicity was confirmed in the original host and in Red Globe grapevines (Peixoto et al., 2007). In the present study we provide further evidence on the identification of three *Amaranthus* strains, collected in a Red Globe area in 2012. Pathogenicity of these strains on grapevine was confirmed. Based on MLSA these strains have 100% identity to the grape strains, confirming the potential of pathovar *viticola* to survive and infect weeds such as *Amaranthus* sp. as alternative hosts. Neem is often employed as windbreaks in vineyards in Brazil and has been described as a natural host in India (Nayudu, 1972). Mango is also grown in the same region in Brazil and can develop symptoms upon inoculation with pathovar *viticola* (Chand and Kishun, 1990). However, natural populations of pathovar *viticola* infecting neem or mango have never been reported. Isolations from neem have been unsuccessful (Peixoto et al., 2007) and whether pathovar *viticola* strains can survive epiphytically and/or infect mango under natural conditions remains unknown.

Genome mining revealed that strain CFBP 7764 had all genes necessary to a *Xanthomonas* strain to sense and move in its environment, to protect itself, and to acquire nutrients. Presence of the different types of secretion systems (T1SS to T6SS) and their numerous effectors confirmed the pathogenic nature of strain CFBP 7764. Comparison of the genomic sequences of the two strains of *X. citri* pv. *viticola* did not show any differences in all these functions but revealed differences mostly in plasmid content. Indeed, the presence of sequences that matched with one or several plasmids was detected in strain CFBP 7764, and the sequences had no orthologs in LMG 965; reciprocally the sequences from one plasmid of LMG 965 had no orthologs in CFBP 7764. However, the sequencing technology used did not allow to obtain a sufficiently high-quality sequence to properly assemble the putative plasmids. Plasmids allow phytopathogenic bacteria to maintain a dynamic, flexible genome and possible advantage in host–pathogen and other environmental interactions (Sundin, 2007).

We proposed a new VNTR scheme, based on the analysis of genomic sequences of two strains representing epidemics from India and Brazil. This scheme could complete the previously proposed *X. citri* pv. *citri* VNTR scheme. Indeed, the VNTRs from the newly proposed scheme were chosen specifically to have different copy numbers between the two sequenced strains in order to enhance the probability to have variable loci in Brazilian vs. Indian strain collections. This scheme encompasses a majority of VNTRs with a short repeat motif (≤7) that should be particularly well suited for epidemiologically related strains as previously mentioned (Pruvost et al., 2014). Furthermore, we designed some VNTRs with a longer repeat motif (up to 16), all together that should allow epidemiological surveys at various scales, with shorter repeat motifs being suited for small to medium spatio-temporal scales and larger ones for global surveillance (Poulin et al., 2015). This study is the first step toward a MLVA scheme suitable for assessing the genetic structure of pathovar *viticola*, which may help to identify inoculum sources and understand how this pathogen disseminated at both local and intercontinental scales. Comparing population diversity of this bacterial pathogen in its native area (India) and invaded regions in Brazil, may contribute to our knowledge of how bacterial plant pathogens emerge and adapt in new environments. Furthermore, the recent availability of two complete genome sequences of pathovar *viticola* (this study, Lima et al., 2017) will improve our understanding of genome diversity and the relationships among strains from different geographical origins.

## Conclusion

In this study, we used sequences of housekeeping genes to confirm the taxonomic status of strains pathogenic on grapevine and *Amaranthus* as members of *Xanthomonas citri* pv. *viticola.* We demonstrated that pathovar *viticola* is a well-defined and monophyletic pathovar, distinct from three other pathovars from India, that affect plants in the Vitaceae family. Based on MLSA, Brazilian strains do not differ from two ancient strains from India. In contrast, eight polymorphic VNTR markers allowed us to assess the genetic structure of the pathogen in Brazil and suggested one introduction event of two genetically distinct groups of strains that lead to adaptation of this pathogen in the country. MLVA showed that Brazilian strains from 1998 and the two ancient Indian strains are not epidemiologically linked. Whole genome comparisons between two strains from India and Brazil, collected within a gap of 43 years revealed new VNTR markers that could be useful to assess diversity at various scales. Our results provided novel information and insights into how this pathogen emerged in Brazil. Validation of this method with a larger collection of strains, especially from India, could be subject of future studies. This is the first report of a MLVA scheme for rapidly assessing diversity in this plant pathogen.

## Author Contributions

MF and M-AJ conceived and designed the study. MF and SB performed the multilocus sequencing analysis. AD and MB performed the genome analysis. PP, MG, MAB, ES, and RM contributed with strain collection, characterization and pathogenicity tests. MF and SC designed and performed the VNTR analysis. MF, M-AJ, AD, and SC wrote and critically reviewed the manuscript. All authors read and approved the final manuscript.

## Conflict of Interest Statement

The authors declare that the research was conducted in the absence of any commercial or financial relationships that could be construed as a potential conflict of interest.

## Footnotes

1 http://147.99.127.226/pub/cfbp/user_tool.php

2 http://eburst.mlst.net/

3 http://insilico.ehu.es/mini_tools/discriminatory_power/index.php
