# Supplementary material for: Xanthomonas citri pv. viticola Affecting Grapevine in Brazil: Emergence of a Successful Monomorphic Pathogen
Source: Front Plant Sci. 2019 Apr 18;10:489. doi: 10.3389/fpls.2019.00489 (PMC6482255; doi:10.3389/fpls.2019.00489)
Supplement: Supplementary file 1 [file Table_1.pdf]

Supplementary Table S1 Probabilities of the Shimodaira-Hasegawa (SH) test of maximum likelihood tree topologies based on the alignments of seven housekeeping genes sequences and the concatenated data set. Values in bold (*P>0.05*) indicate that the tree topologies are congruent

Phylogenetic tree	*atpD*	*dnaK*	*Efp*	*fyuA*	*glnA*	*gyrB*	*rpoD*	Concatenated dataset
atpD	*	0.000	0.000	0.000	0.000	0.000	0.000	0.000
dnaK	0.000	*	**0.256**	0.000	0.000	0.000	0.001	0.000
efp	0.000	0.000	*	0.000	0.000	0.000	0.000	0.000
fyuA	0.000	0.000	0.007	*	0.000	0.000	0.000	0.000
glnA	0.000	0.000	0.004	0.000	*	0.000	0.000	0.000
gyrB	0.000	0.000	0.000	0.000	0.000	*	0.000	0.000
rpoD	0.000	0.000	0.000	0.000	0.000	0.000	*	0.000
Concatenated dataset	**0.288**	**0.552**	**0.440**	**0.115**	0.011	**0.333**	0.038	*
