# Supplementary material for: Xanthomonas citri pv. viticola Affecting Grapevine in Brazil: Emergence of a Successful Monomorphic Pathogen
Source: Front Plant Sci. 2019 Apr 18;10:489. doi: 10.3389/fpls.2019.00489 (PMC6482255; doi:10.3389/fpls.2019.00489)
Supplement: Supplementary file 2 [file Table_2.pdf]

Supplementary Table S2 Different kind of ISs present in the genomes of strains CFBP 7764 and LMG 965 (results of tBLASTNs)

		CFBP 7767			LMG 965		
IS name	size bp	% length	% identity	locus tag	% length	% identity	locus tag
IS112/IS5	883	4	83	XAVTCFBP7764_c04180	4	83	CBZT010000002.454
IS1389/IS3	1214	99	96	XAVTCFBP7764_ak00020	99	96	CBZT010000036.5
IS1421/IS5	864	3	89	XAVTCFBP7764_d03440	3	89	CBZT010000003.164
IS1477 orfA	204				15	87	CBZT010000003.195
IS1478/IS5	1207	8	87	XAVTCFBP7764_b02060	8	87	CBZT010000005.197
IS150/IS3	1443	2	85	XAVTCFBP7764_e00590	2	85	CBZT010000021.28
IS1595/IS1595	1072	53	86	XAVTCFBP7764_d02330	53	86	CBZT010000003.56
ISXac2/IS3	1195	54	99	XAVTCFBP7764_bb00020			
ISXac3/IS3	1234	43	100	XAVTCFBP7764_j00280	100	100	CBZT010000042.1
ISXac4/IS3	1202	100	96	XAVTCFBP7764_f00530	100	96	CBZT010000022.53
ISXal2	948	3	86	XAVTCFBP7764_c02220	3	86	CBZT010000002.268
ISXal3 orfA	267	55	97	XAVTCFBP7764_f00540	55	97	CBZT010000022.54
ISXal5	798	4	83	XAVTCFBP7764_g00680	3	88	CBZT010000022.36
ISXal6 orfB	423	5	100	XAVTCFBP7764_c04730	5	100	CBZT010000002.504
ISXal7 orfA	294	16	81	XAVTCFBP7764_bm00010	16	81	CBZT010000042.2
ISXc8/IS3	1227	9	88	XAVTCFBP7764_c03080			
ISXcc1 orfB/IS3	768	63	89	XAVTCFBP7764_a05710	63	89	CBZT010000001.112
ISXo15/IS701	1473	3	82	XAVTCFBP7764_l00570	3	82	CBZT010000015.56
ISXo3/IS5	844	3	88	XAVTCFBP7764_m00060	3	88	CBZT010000011.115
ISXo7/IS630	1114	3	85	XAVTCFBP7764_g00830	3	85	CBZT010000009.14
ISXoo12/unknown	447	6	89	XAVTCFBP7764_d02900	6	89	CBZT010000003.110
ISXoo2	1059	3	81	XAVTCFBP7764_b03450	3	81	CBZT010000004.48
ISXoo6/IS5	1170	93	80	XAVTCFBP7764_p00270	93	80	CBZT010000011.26
