# Supplementary material for: Xanthomonas citri pv. viticola Affecting Grapevine in Brazil: Emergence of a Successful Monomorphic Pathogen
Source: Front Plant Sci. 2019 Apr 18;10:489. doi: 10.3389/fpls.2019.00489 (PMC6482255; doi:10.3389/fpls.2019.00489)
Supplement: Supplementary file 4 [file Table_4.pdf]

Supplementary Table S4 The eight *Xanthomonas citri* pv. *citri* (Xcc) VNTRs used in this study found in the *X. citri* pv. *viticola* strains CFBP 7764 and LMG 965 (CFBP 7660) genome sequences and the obtained copy numbers after sequencing the PCR- amplified loci for both strains

VNTR loci	Xcc repeat sequence	Whole genome sequencing		Amplicon sequencing	
		Xcvt repeat sequence	Copy number predicted CFBP 7764 / LMG 965	Xcvt repeat sequence	Copy number obtained CFBP 7764 / LMG 965
XL1	TTCCCCA	TTCCCCA	4/4	TTCCCca (gc) (ag)	9/10
XL3	TTTCCGA	TTTCCGA	10/6	TTtCCGA/ TTcCCGA	13/9
XL4	CCCGAAT	CCCGAAT	10/6	CCCGAaT/ CCCGAtT	11/6
XL5	TTCCCGG	aTCCCG**A**	8/10	aTCCCG**A** /tTCCCG**A**	8/10
XL6	CCGATTC	CCG**c**TTC	24/24	CCGcTTC /CCGaTTC	24/24
XL8	CGGGAAT	CGGGAAT	2/3	CGGgAAT/CGGaAAT	5/13
XL13	CGGGAAT	CG**T**GAAT	8/8	CG**T**GAAT	7/8
XL15	CGAATCC	**G**GAATC**G**	10.4/5.4	CGAtTCC/ CGAaTCC	10/5
