# Supplementary material for: Xanthomonas citri pv. viticola Affecting Grapevine in Brazil: Emergence of a Successful Monomorphic Pathogen
Source: Front Plant Sci. 2019 Apr 18;10:489. doi: 10.3389/fpls.2019.00489 (PMC6482255; doi:10.3389/fpls.2019.00489)
Supplement: Supplementary file 5 [file Table_5.pdf]

Supplementary Table S5 *In silico* positioning of the eight Xcc VNTRs used in the study in the *X. citri* pv. *viticola* strains CFBP 7764 and LMG 965 genome sequences.

			CFBP 7764			LMG 965		
VNTR loci	Xcc repeat sequence	Xcvt repeat sequence	Contig nb.	Start position	End position	Contig nb.	Start position	End position
XL1	TTCCCCA	TTCCCCA	Xavit-CFBP7764-G105	168697	168448	NZ_CBZT010000019.1	51388	51388
XL3	TTTCCGA	TTTCCGA	Xavit-CFBP7764-G117	4262	3960	NZ_CBZT010000002.1	50853	50853
XL4	CCCGAAT	CCCGAAT	Xavit-CFBP7764-G104	292652	292440	NZ_CBZT010000003.1	134267	134267
XL5	TTCCCGG	aTCCCG**A**	Xavit-CFBP7764-G109	143547	143356	NZ_CBZT010000018.1	77570	77570
XL6	CCGATTC	CCG**c**TTC	Xavit-CFBP7764-G101	327378	327089	NZ_CBZT010000001.1	464443	464443
XL8	CGGGAAT	CGGGAAT	Xavit-CFBP7764-G107	169609	169380	NZ_CBZT010000009.1	87928	87928
XL13	CGGGAAT	CG**T**GAAT	Xavit-CFBP7764-G104	328157	327946	NZ_CBZT010000003.1	169738	169738
XL15	CGAATCC	**G**GAATC**G**	Xavit-CFBP7764-G101	261230	261671	NZ_CBZT010000001.1	529839	529839
